# Supplementary material for: A data-driven Boolean model explains memory subsets and evolution in CD8+ T cell exhaustion
Source: NPJ Syst Biol Appl. 2023 Jul 31;9:36. doi: 10.1038/s41540-023-00297-2 (PMC10390540; doi:10.1038/s41540-023-00297-2)
Supplement: Supplementary file 1 — Supplementary material [file 41540_2023_297_MOESM1_ESM.pdf]

A data-driven Boolean model explains memory subsets and evolution in CD8+ T cell exhaustion

Geena V. Ildefonso,[1] Stacey D. Finley[1,2,3*]

[1] Alfred E. Mann Department of Biomedical Engineering, University of Southern California, Los Angeles, California, USA
[2] Department of Quantitative and Computational Biology, University of Southern California, Los Angeles, California, USA
[3] Mork Family Department of Chemical Engineering and Materials Science, University of Southern California, Los Angeles, California, USA

[*]Correspondence: sfinley@usc.edu

SUPPLEMENTARY FIGURES

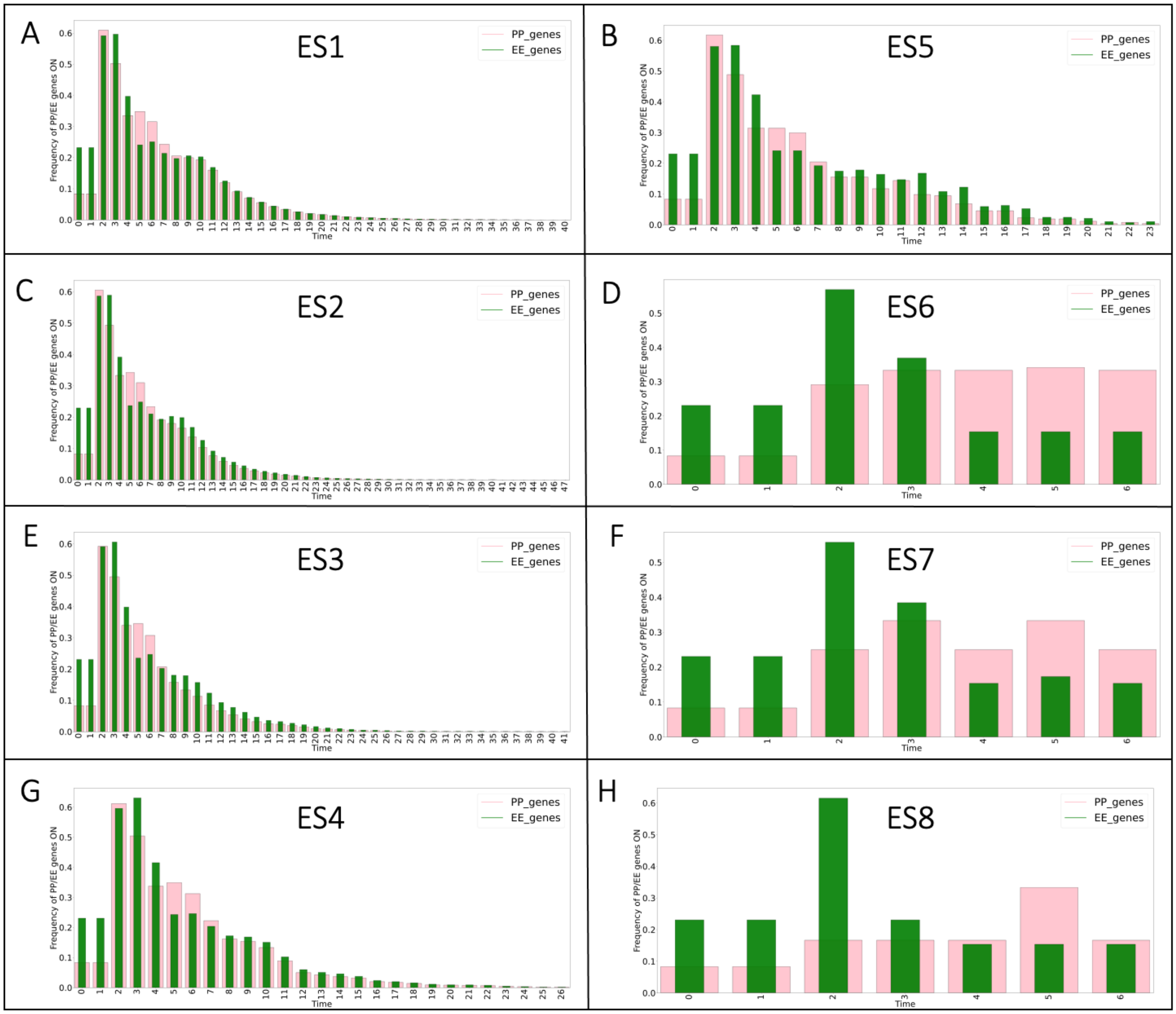

Supplementary Figure 1. Fractional activation profiles following initial T cell activation. (A-H) Fraction of PP and EE gene activation for all simulations over time for each end state.

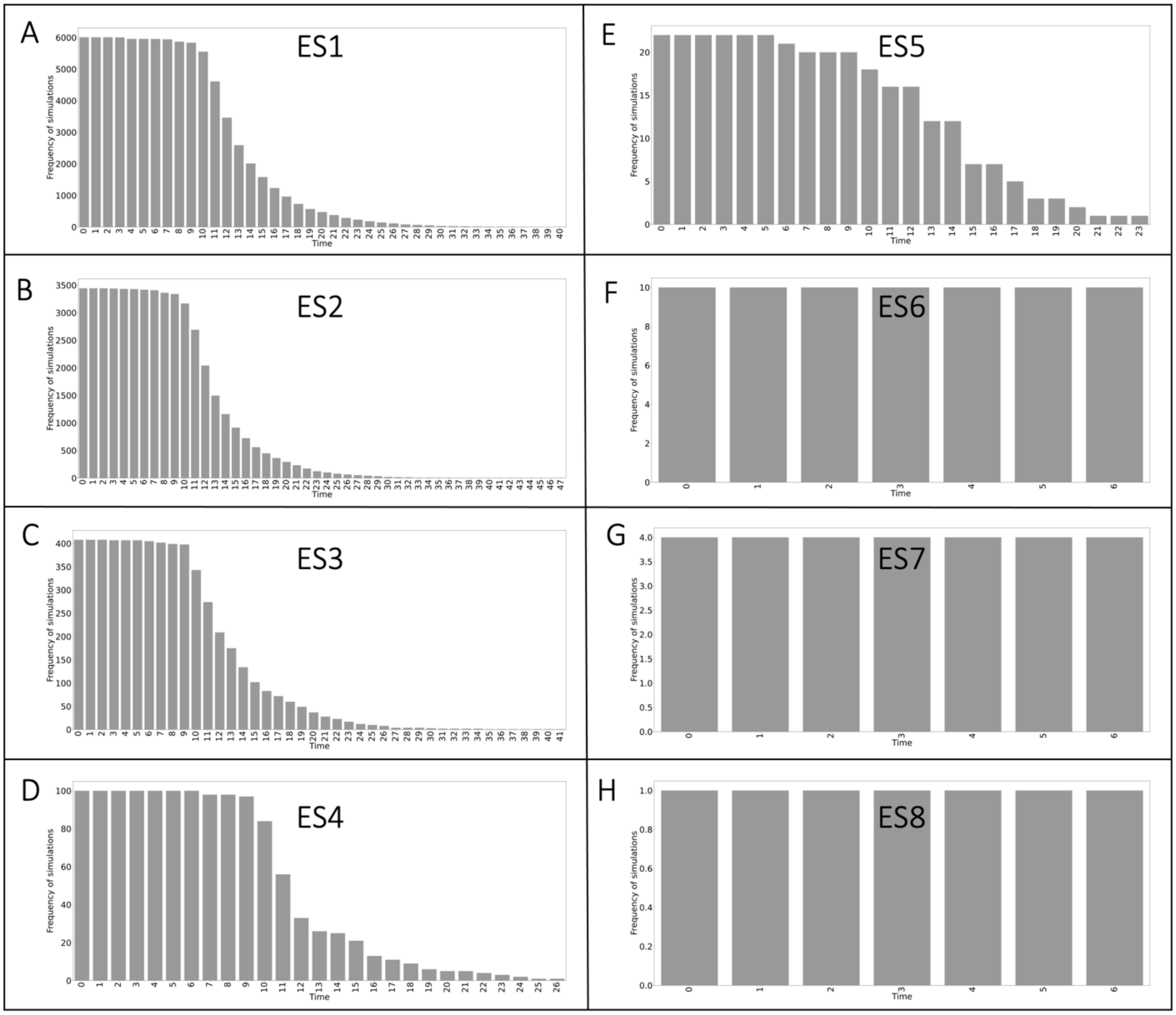

Supplementary Figure 2. Number of simulations that have not reached their terminal end state. (A-H) Frequency distributions for each end state following initial T cell activation.

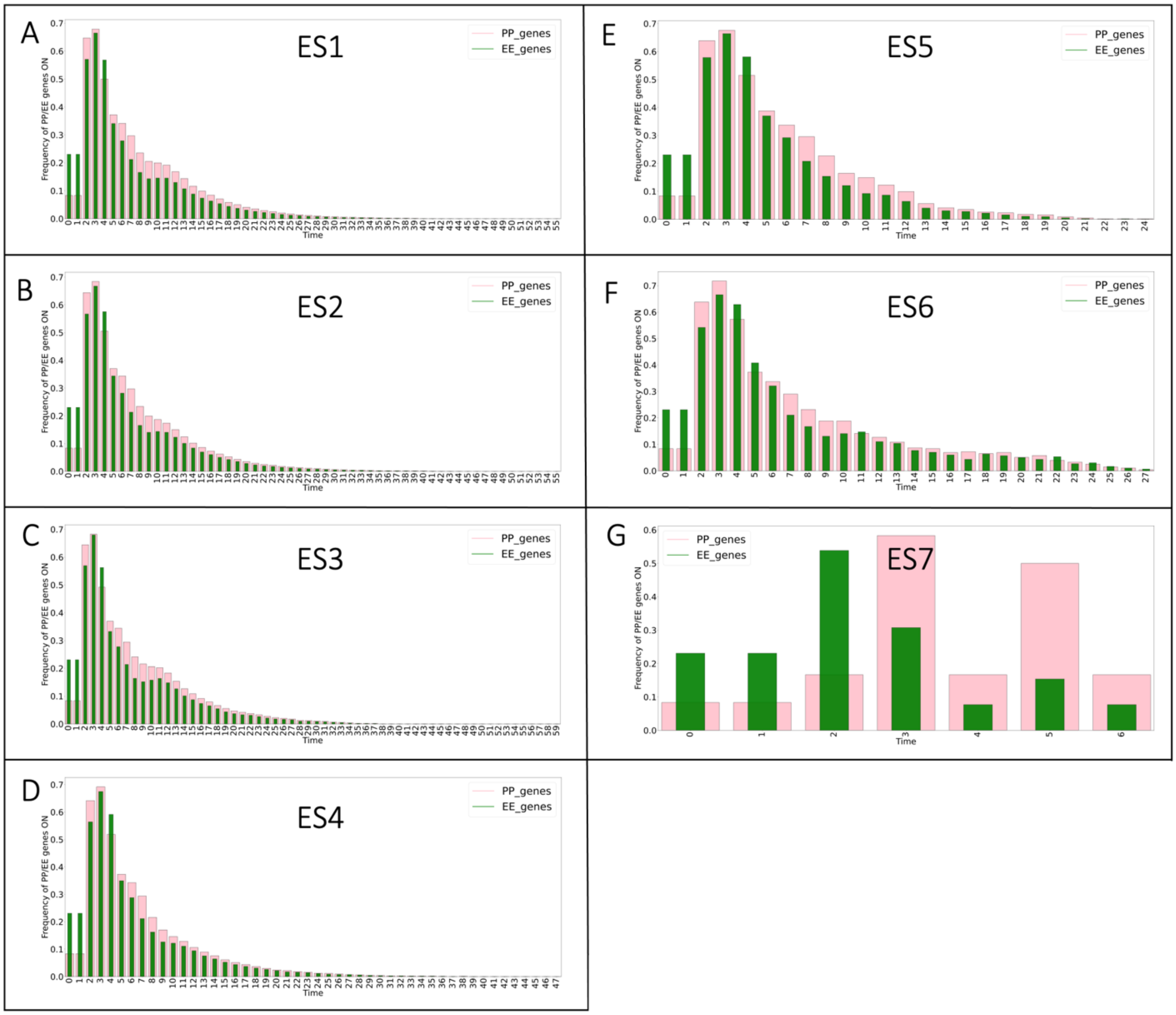

Supplementary Figure 3. Fractional activation profiles following initial T cell activation with PD1 blockade. (A-G) Fraction of PP and EE gene activation for all simulations over time for each end state with PD1 blockade

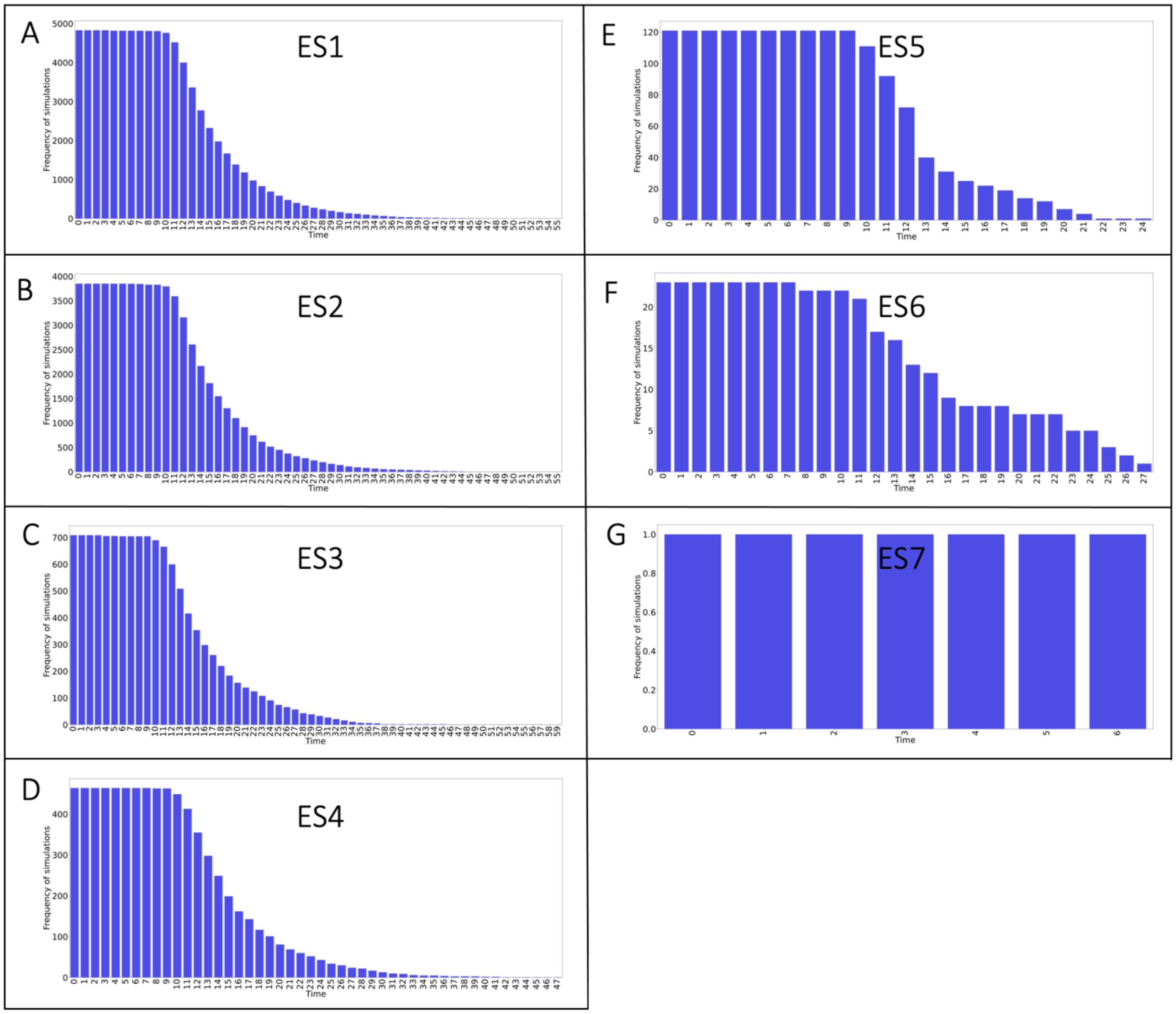

Supplementary Figure 4. Number of simulations that have not reached their terminal end state. (A-H) Frequency distributions for each end state with PD1 blockade.

SUPPLEMENTARY TABLES

Supplementary Table 1. Set of rules for the Boolean model[1]. Rules 1-22 represent the WT Boolean model simulations, rule 23 is the indirect PD1 blockade. 'S' represents the network state. The symbols '&', '|' and '!' represent logical AND, OR and NOT operators respectively. 'prevS1' and 'prevS2' are the states of the network at 1 and 2 steps earlier.

Rule number	Rule
1	(S['TCR'] & !S['PD1']) → S['TCR']
2	S['TCR'] → S['IL2'] = S['IL21'] = S['IL12']
3	(!S['IL2'] & !S['AKT']) & (prevS2['TCR'] | prevS2['STAT3']) → S['FOXO1']
4	(S['TCR'] & !S['PD1']) → S['AKT']
5	(S['NFATC1'] | S['PD1']) → S['NR4A1']
6	((prevS2['AP1'] & prevS1['AP1'] & S['AP1']) | S['FOXO1'] | S['NFATC1']) → S['PD1']
7	(prevS1['AP1'] | prevS2['AP1'] | S['AP1']) & !S['BCL6'] → S['AP1.DNA']
8	(S['BATF.IRF4'] | S['IL2'] | S['AP1.DNA']) → S['BLIMP1'
9	(S['NFkB'] & !S['NR4A1']) → S['IRF4']
10	((S['FOXO1'] | S['BATF'])) & ! S['IRF4'] → S['BCL6']
11	S['BCL6'] → S['TCF1']
12	(S['BATF'] & S['TCR']) → S['BATF.IRF4']
13	(S['IL12'] | S['IL21']) → S['BATF']
14	(S['IL2'] & !S['FOXO1']) → S['IL2']
15	(prevS2['NFATC1.med'] | prevS1['NFATC1']) → S['NFATC1']
16	prevS2['NFATC1.lo'] → S['NFATC1.med']
17	prevS2['NFATC2'] → S['NFATC1.lo']
18	(S['NFATC2'] & S['AP1'] & !S['TCF1']) → S['IFNg']
19	S['TCR'] → S[c('NFkB', 'NFATC2', 'AP1')]
20	1→ S['AP1']
21	S['AKT'] → S['mTOR'] → S['glycolysis']
22	prevS1['IL21'] → S['STAT3']
23 (PD1 inhibition)	((prevS2['AP1'] & prevS1['AP1'] & S['AP1']) | S['FOXO1']) → S['PD1']

REFERENCES

1. Bolouri, H. *et al.* Integrative network modeling reveals mechanisms underlying T cell exhaustion. *Sci. Reports 2020 101* **10**, 1–15 (2020).
